# Draft Genome Sequence of JVAP01^T^, the Type Strain of the Novel Species *Acinetobacter dijkshoorniae*

**DOI:** 10.1128/genomeA.01480-16

**Published:** 2017-01-12

**Authors:** Dietmar Fernández-Orth, Clara Cosgaya, Murat Telli, Noraida Mosqueda, Marta Marí-Almirall, Ignasi Roca, Jordi Vila

**Affiliations:** a Department of Clinical Microbiology and ISGlobal, Barcelona Ctr. Int, Health Res. CRESIB, Hospital Clínic, Universitat de Barcelona, Barcelona, Spain; b Department of Clinical Microbiology, School of Medicine, Adnan Menderes University, Aydin, Turkey

## Abstract

Here, we report the draft genome sequence of the type strain of *Acinetobacter dijkshoorniae*, a novel human pathogen within the *Acinetobacter calcoaceticus–Acinetobacter baumannii* (ACB) complex. Strain JVAP01^T^ has an estimated genome size of 3.9 Mb, exhibits a 38.8% G+C content, and carries a plasmid with the *bla*_NDM-1_ carbapenemase gene.

## GENOME ANNOUNCEMENT

The *Acinetobacter calcoaceticus–Acinetobacter baumannii* (ACB) complex currently comprises six different *Acinetobacter* species, the environmental *A. calcoaceticus* and five *Acinetobacter* species that are potential human pathogens, that is, *A. baumannii*, *A. pittii*, *A. nosocomialis*, *A. seifertii*, and *A. dijkshoorniae*, the latter two only recently discovered (1). Members of the ACB complex are virtually undistinguishable from a biochemical standpoint and can only be differentiated by means of molecular methods (2, 3). Hospital outbreaks are mostly attributed to *A. baumannii*, whose innate ability to accumulate multiple antibiotic resistance mechanisms is greatly feared (4). The advent of more reliable identification methodologies, however, has shown an alarming abundance of all other species in the clinical setting, as well as their potential to bear resistance mechanisms to last resort antibiotics (5, 6). Here we report the draft genome sequence of strain JVAP01^T^ (CECT 9134^T^, LMG 29605^T^), the type strain of *Acinetobacter dijkshoorniae* that was recovered in 2009 from a urine sample in Turkey. JVAP01^T^ produces the NDM-1 metallo-β-lactamase and is resistant to β-lactam antibiotics and kanamycin (7).

Genomic DNA was extracted from cultured bacteria and an Illumina library was generated following Nextera XT (Illumina, Inc., San Diego, CA, USA) manufacturer’s protocol with paired-end libraries (2 × 150). Sequencing was performed in an Illumina MiSeq system. *De novo* assembly was performed using Velvet version 1.2.10 in conjunction with the Velvet optimizer (http://bioinformatics.net.au/software.velvetoptimiser.shtml), ABySS v1.5.2 and Spades v3.5.0 (8–10). Contigs for all assemblers were joined using CISA v1.3 (11). CISA contigs below 200 nucleotides were discarded to yield a total of 92 contigs with a 90-fold coverage. The draft genome comprised a total assembly length of 3,858,459 bp and the G+C content was in accordance with that of *Acinetobacter* spp., at 38.8%.

The sequence of the 47 kilobase plasmid (pNDM-JVAP01) containing the *bla*_NDM-1_ gene and a type VI secretion system was previously published (7).

All 92 contigs and the plasmid were further annotated using the RAST server (12), which predicted 3,599 coding sequences (CDS), 26 rRNAs, and 134 tRNAs in the genome. In order to classify the antibiotic resistance gene pools, Resfinder v2.1 with a threshold of 85% identity and a minimum length of 40% was used (13). Results showed the presence of the *bla*_NDM-1_ and *aphA6* genes, described previously and conferring resistance to most β-lactams and aminoglycosides, respectively (7), but also of the *bla*_ADC_- and *bla*_OXA-213_-family genes, respectively encoding an *Acinetobacter-*derived cephalosporinase and a class D oxacillinase, both of chromosomal location and conferring resistance to β-lactams. PathogenFinder v1.1 was used for the prediction of bacterial pathogenicity (14). Results revealed this species as human pathogen (83.8% probability) matching common sequences with 24 pathogenic families. Those, among others, were from *A. baumannii* ATCC 17978, AB0057, ACICU, and AYE strains.

### Accession number(s).

This whole-genome shotgun project has been deposited in DDBJ/ENA/GenBank under the accession no. LJPG00000000 and KM923969 for its associated plasmid. The version described in this paper is LJPG00000000.1.
